# Cognitive and neuropsychomotor development in craniosynostosis: an evaluation of the most affected functions

**DOI:** 10.1007/s00381-026-07127-w

**Published:** 2026-01-29

**Authors:** Jéssica Luchi Ferreira, Igor José Nogueira Gualberto, Mariani Da Costa Ribas, Michele Madeira Brandão, Cristiano Tonello

**Affiliations:** 1 Department of Craniofacial Surgery, Hospital for Rehabilitation of Craniofacial Anomalies - University of São Paulo, Bauru, Brazil São Paulo; 2 Medical School, University of São Paulo, São Paulo, Brazil São Paulo; 3 Medical School of Bauru, University of São Paulo, Bauru, Brazil São Paulo

**Keywords:** Craniosynostoses, Neuropsychology, Cognitive function, Neurodevelopmental disorders

## Abstract

**Background:**

The physical manifestations of craniosynostosis are well-documented, but its impact on neurodevelopment and cognitive functions remains under-researched. Evaluating neurodevelopment and cognition is essential to establish baseline cognitive functions in surgical procedures, guiding therapeutic and rehabilitation strategies post-surgery. This study aims to characterize the cognitive profile and neuropsychomotor development in individuals with craniosynostosis, identifying the most affected functions.

**Methods:**

This study was a retrospective, observational, cross-sectional chart review that analyzed 65 individuals with craniosynostosis (39 syndromic, 26 isolated) who underwent neuropsychological assessments. Five key instruments were used to assess neuropsychomotor development and cognitive functions across different age groups.

**Results:**

Significant neuropsychological differences were found between syndromic and isolated groups (*p* = 0.026). Key findings include the following: 68.4% of syndromic patients showed altered neuropsychological results; 40.7% of the isolated group demonstrated neuropsychological variations; language skills most frequently affected; fluid intelligence deficits in 45.5% of patients; motor gross impairments in 24.0%, predominantly in the isolated group. A weak negative correlation was observed between surgery age and cognitive improvement (*r* = −0.32), with early surgery (0–1 year), mixed outcomes, and later surgery (3 + years), poorer cognitive results.

**Conclusion:**

Early interventions are crucial for reducing developmental delays, though individual variability and syndromic conditions significantly influence outcomes. Focusing on early intervention, targeted rehabilitation, and smooth school transition can optimize cognitive development and manage potential delays.

**Supplementary Information:**

The online version contains supplementary material available at 10.1007/s00381-026-07127-w.

## Introduction

Craniosynostosis may occur as part of syndromic conditions or as isolated single suture cases, with prevalence estimates of up to 1:30,000 for syndromic forms and approximately 1:2000–2500 for isolated cases [1, 2]. Mutations in genes such as FGFR1, FGFR2, FGFR3, and TWIST1 underlie many syndromic presentations (Pfeiffer, Crouzon, Apert, Saethre-Chotzen) and contribute to multisystem phenotypes beyond cranial deformity [3].

Historically regarded primarily as a cosmetic condition, craniosynostosis is now studied for potential impacts on cognition, language, visuomotor integration, and motor development, and for how neurosurgical timing and technique may influence these outcomes [4–7].

Longitudinal and multicenter cohorts (for example, the Infant and Child Learning Project) have documented lower scores in cognition, language, and academic functioning in some children with single suture craniosynostosis compared with controls [8–11], but systematic reviews note substantial heterogeneity across studies driven by sample composition (isolated versus syndromic), assessment instruments, age at evaluation, and surgical factors, limiting definitive conclusions [12–14].

Cognition encompasses attention, memory, reasoning, and related processes [15], while neuropsychomotor development integrates cognitive, motor, language, and visuospatial functions and their coordination [16]. Evidence of cognitive, language, and motor impairments has been reported in both isolated and syndromic cases [5, 17, 18]; because isolated craniosynostosis has a multifactorial etiology and syndromic cases carry additional systemic risks, including these groups together, enables characterization of phenotypic variability.

Preoperative neurodevelopmental assessment (NPA) establishes a baseline to guide rehabilitation and follow-up [19]. This study, therefore, aims to characterize the cognitive profile and neuropsychomotor development of individuals with craniosynostosis and to identify the functions most affected.

Considering the broad spectrum of neuropsychological features in craniosynostosis, this study aims to compare the neuropsychological profile of individuals with syndromic craniosynostosis (SC) and isolated/non-syndromic craniosynostosis (IC) using standardized assessments to identify the domains most frequently altered.

## Methods

This study is a retrospective cross-sectional analysis approved by the Institutional Review Board (6.682.904) involving individuals with isolated and syndromic craniosynostosis clinics assessed by the hospital genetic service through imaging exams at our centre between 2017 and June 2024. Patients who did not undergo any NPA were excluded. All ethical precautions were carefully followed.

From 71 eligible patients, six were excluded (four lacked any NPA, one CPM-RAVEN profile showed insufficient internal consistency, and one assessment used a non-standardized instrument), yielding a final analytic sample of 65 participants organized into syndromic (SC, *n* = 39) and isolated/non-syndromic (IC, *n* = 26) groups. The isolated group comprised primarily single-suture cases (sagittal, metopic, unicoronal), with non-syndromic multisuture cases retained only after clinical and genetic evaluation excluded a syndromic etiology; suture involvement was recorded for all cases, and small subgroup sizes are reported descriptively when inferential testing was inappropriate.

### Assessing neuropsychomotor development

#### Dimensional inventory of child development assessment (IDADI)

Evaluate child development from birth to 72 months through parental reporting, covering cognitive, motor (gross and fine), communication and language (receptive and expressive), socioemotional, and adaptive behaviour domains. Scores follow a standard of 2 points for “Yes,” 1 point for “Sometimes,” and 0 points for “Not yet” for the described skills [20].

#### Behaviour developmental scale of gesell and amatruda (BDSGA)

Assess child development from birth to 36 months across five domains: motor (gross and fine), language, personal-social, and adaptive behaviour. This evaluation allows the calculation of a developmental quotient (DQ), which correlates chronological age with developmental age. The DQ can estimate infant development, with an average described as 100 by the BDGSA [21].

### Assessing cognitive function

#### Wechsler abbreviated scale of intelligence (WASI)

This assesses the intelligence of individuals aged 6 to 89 years old by measuring performance (PIQ), verbal (VIQ), and Full-Scale Intelligence Quotient (FSIQ). The instrument comprises four subtests: two verbal (vocabulary and similarities)—quantify word knowledge and verbal reasoning—and two performances (block design and matrix reasoning)—analyze visuospatial skills; FSIQ is calculated considering PIQ and VIQ results. The average standard score is 100 [22].

#### Wechsler intelligence scale for children (WISC-IV)

This assesses the intelligence of individuals aged 6 to 16 years and 11 months old by measuring performance (Perceptual Organization Index – POI), verbal (Verbal Comprehension Index – VCI), Working Memory Index (WMI), and Processing Speed Index (PSI). The instrument comprises 15 subtests. FSIQ is calculated considering the results from POI, VCI, WMI, and PSI [23].

#### Raven progressive matrices (CPM-RAVEN)

A nonverbal measure of fluid intelligence, the test assesses the ability to solve novel reasoning problems—an important component of general intelligence (factor g)—in children aged approximately 5 to 11.5 years. It consists of 36 items presented in a missing-piece puzzle format, spread across three different scales and arranged in increasing difficulty. Scores were converted to Brazilian normative percentiles for interpretation [24].

#### SON-R 2 ½–7 [a]—non-verbal intelligence test

The SON-R 2½–7[a] comprises four subtests that yield three scales—Execution (SON-EE), Reasoning (SON-ER), and Global (SON-QI)—and assesses nonverbal reasoning, spatial perception, and visual-constructive abilities in children aged 2.5–7 years; it does not evaluate receptive or expressive language [25].

By NPA, we refer to the applied procedure from the field of neuropsychology used to characterize cognitive, emotional, and behavioural functioning; as a tool of neuropsychology, the NPA combines standardized neuropsychological tests with systematic clinical observation and functional information, and all primary outcome data reported in the manuscript are derived from these test results [26].

All NPAs were conducted by the head psychologist, with residents participating under supervision, as part of routine multidisciplinary clinic visits; assessments were provided free of charge to families. Clinical evaluation by the multidisciplinary craniofacial team was performed both before and after surgery and, when indicated, included CT and MRI imaging; ophthalmological assessment was incorporated into both preoperative and postoperative evaluations. As these evaluations formed part of standard clinical care, no separate clinical consent form was required for their performance; however, informed consent was obtained from participants or their guardians for inclusion in the research study and for use of anonymized data.

When the caregiver’s report was unreliable, items ordinarily obtained by parents were administered or directly observed with the child to reduce bias. Instruments were chosen by age and psychometric suitability (nonverbal tests preferred for very young children or when language impairment was suspected), and all scores were converted to Brazilian normative percentiles. Classification used uniform bands: 25th–75th percentile = expected range, < 25th = at risk/delayed, and ≈2nd–3rd percentile (or instrument equivalent) = impaired, applied across SON-R, WISC-IV, WASI, CPM-RAVEN, and IDADI. Statistical analyses were descriptive and categorical (chi-square, Fisher exact, cross-tabulation), with significance set at *p* ≤ 0.05 and analyses performed in Jamovi v2.5 [27].

Categorical comparisons between independent groups were performed with chi-square tests; cross-tabulations examined outcome distributions, and descriptive analyses summarized the data.

## Results

A total of 65 individuals with craniosynostosis were included in the study and were classified as syndromic (SC, *n* = 39) or isolated (IC, *n* = 26) (see Table 1 for diagnostic composition). Seven participants remained unoperated and are described separately.

**Table 1:** Descriptive values and comparative analysis of the groups regarding the diagnosis

		***N***	Percent	Diagnosis	***N***	Percent
Groups	Syndromic (SC)	39	60	Apert	13	33.33%
				Crouzon	21	53.85%
				CFND	3	7.69%
				Pfeiffer	1	2.56%
				Saethre-Chotzen	1	2.56%
	Isolated (IC)	26	40	Bicoronal Suture	5	19.23%
				Sagittal Suture	14	53.85%
				Unicoronal Suture	4	15.38%
				Metopic Suture	3	11.54%

*SC* syndromic craniosynostosis, *CFND* craniofrontonasal dysplasia, *IC* isolated craniosynostosis

The battery of instruments varied by age and clinical indication: BDSGA was administered most frequently (*n* = 25; 38.46%), followed by WASI (*n* = 19; 29.23%), CPM-Raven (*n* = 11; 16.92%), IDADI (*n* = 8; 12.31%), SON-R (*n* = 1; 1.54%), and WISC-IV (*n* = 1; 1.54%) (Table 2). Because participants span infancy through adulthood, instruments and subscales differ by age group; all scores were converted to percentile units, and domains < 25th percentile range were coded as “altered” for descriptive comparisons across instruments and groups.

**Table 2:** General characteristics of the sample according to assessment instrument, evaluation timing, type of surgery, and economic class

	Variables	***N***	Percent
Instrument	Gesell	24	36.9%
	IDADI	8	12.3%
	CPM-RAVEN	12	18.5%
	SON-R 2 1/2 −7 [a]	1	1.5%
	WASI	19	29.2%
	WISC-IV	1	1.5%
Evaluation timing	Preoperative	13	20.0%
	Postoperative	52	80.0%
Surgery technique	Cranioplasty	20	30.7%
	Cranioplasty w/distraction	1	1.5%
	Endoscopic suturectomy	2	3.1%
	Frontofacial monobloc advancement (FFMA)	8	12.3%
	Fronto-orbital advancement	2	3.1%
	Le Fort III	9	13.9%
	Le Fort III and FFMA*	1	1.5%
	Not operated	8	12.3%
	Posterior Vault Decompression	9	13.9%
	Posterior Vault Decompression and FFMA	4	6.2%
	Posterior Vault Decompression and Le Fort III	1	1.5%
Economic class*	US$ 342.60	16	12.7%
	US$ 342.61–913.60	30	46.0%
	US$ 913.61–2,284	15	23.0%
	US$ 2,284.01–4,568	1	1.5%
	US$ > 4,568	3	4.61%

Source: Created by the author, 2025. *FFMA* frontofacial monobloc advancement. *The socioeconomic strata in Brazil are defined based on the minimum wage (US$ 228.40), as established by the Brazilian Association of Market Research Companies

Of the 11 participants assessed with CPM-RAVEN, 5 (45.5%) showed deficits in fluid intelligence; of the 8 assessed with IDADI, 2 (25.0%) showed expressive-language alterations; and among those assessed with infant/preschool instruments, BDSGA most often indicated gross-motor and adaptive-behaviour alterations. Domain-level observations are reported descriptively and summarized in Table 3 and Graph 1.

**Table 3:** Distribution of participants scoring below normative thresholds across assessments

Assessment tool	Domain	*N*	Percent
IDADI	Cognitive	0	0.0%
	Gross motor skills	1	12.5%
	Fine motor skills	1	12.5%
	Communication	0	0.0%
	Receptive language	0	0.0%
	Expressive language	2	25.0%
	Socio-emotional	0	0.0%
	Adaptive behaviour	1	12.5%
WASI	FSIQ*	8	42.1%
	PIQ	7	36.8%
	VIQ	11	42.0%
BDSGA	Gross motor skills	5	24.0%
	Fine motor skills	4	16.0%
	Adaptive behaviour	6	24.0%
	Language	5	20.0%
	Personal-social	6	24.0%
CPM-RAVEN	Altered fluid intelligence	5	45.5%

Source: Created by the author, 2025. *FSIQ* Full-Scale Intelligence Quotient, *PIQ* Performance Intelligence Quotient, *VIQ* Verbal Intelligence Quotient. Reference values: Scores ≤ 25th percentile were coded as below the normative range

Motor (gross and fine) and communication domains were among the most frequently affected across instruments; VIQ-related weaknesses were prominent among older patients tested with WASI/WISC instruments, and CPM-RAVEN identified fluid-reasoning deficits in a substantial proportion of those tested (Graph 1).

The BDSGA most frequently indicated impairments in gross motor skills and adaptive behaviour (24% each; *n* = 5); one of these patients belonged to the IC group and also presented language impairment. The SON-R 2½–7 was administered to a single participant (Crouzon, age 4), whose subscale scores were within the average range; no abnormalities were observed (Table 2).

Motor impairments were observed in gross motor (24%, *n* = 6) and fine motor (15.5%, *n* = 5) domains; the odds ratio (0.1021) shows this impairment is less likely in the SC group (5.1%) than in the IC group (34.6%). Fisher’s exact test indicated a significant difference in motor-skill alterations between SC and IC groups (*p* = 0.0047). 36.8% (*n* = 7) of patients assessed with the WASI demonstrated PIQ deficits; all these individuals were diagnosed with Crouzon or Apert syndrome.

Comparison between SC and IC groups for language impairment (26.15%) using Fisher’s exact test did not show a statistically significant difference (*p* = 0.2656). The unadjusted odds ratio for language impairment in syndromic versus isolated cases was 2.10, suggesting higher odds in the SC group; however, this effect did not reach statistical significance.

Adaptive-behaviour impairments affected 21.2% (*n* = 7; 5 isolated) and personal-social deficits 24% (*n* = 6; two syndromic); no socio-emotional alterations were detected, and the IC group was disproportionately represented among these deficits. Fisher’s exact test indicated no statistically significant difference; the odds ratio was 0.227, with a prevalence of 5.1% in the SC group versus 19.2% in the IC group.

Personal-social skills impairment in 24% (2 SC group) approached significance results for Fisher’s exact test (*p* = 0.0514); the odds ratio (0.1802) corresponds to prevalences of 5.1% in SC versus 23.1% in IC.

Using age at first cranial surgery to assign reviewer bands (with non-operated participants described distinctly), the cohort was distributed across the surgical-timing groups as reported in Table 4 and Graph 2. Ages at first cranial surgery and at NPA are reported as completed years and are summarized as mean ± SD and observed range: mean age at first surgery 6.00 ± 8.46 years (range 0.25–43 years) and mean age at NPA 6.71 ± 8.10 years (range 0.17–40 years). A formal standardized pre/post assessment protocol was implemented in 2017; overall, 52 participants underwent postoperative NPA, and 13 underwent preoperative NPA, of whom 12 had paired pre- and postoperative assessments used for categorical case-level analyses.

**Table 4:** Descriptive values with age at surgery and NPA

	Mean	SD	Max	Min
Age of surgery	6.00 years	8.46 years	43.00 years	3 months
Age of NPA	6.71 years	8.10 years	40.00 years	2 months

The cohort distributed as follows: 0–12 months (*N* = 21)—1 Pfeiffer, 1 Crouzon, 2 Apert, 4 coronal suture, 2 unilateral coronal, 2 metopic, 9 sagittal; > 1–3 years (*N* = 14)—5 Crouzon, 2 craniofrontonasal dysplasia, 2 unilateral coronal, 1 metopic, 4 sagittal; 4–6 years (*N* = 5)—3 Crouzon, 2 Apert; 7–12 years (*N* = 7)—3 Crouzon, 3 Apert, 1 sagittal; 13–18 years (*N* = 8)—4 Crouzon, 4 Apert; 19–43 years (*N* = 3)—2 Crouzon, 1 Apert. Seven participants remain unoperated and are reported separately (*N* = 7, 3 Crouzon, 1 Apert, 1 unilateral coronal, 1 Saethre-Chotzen, 1 coronal suture)—Graph 2.

Likewise, NPA results between the two groups were compared. The chi-square test was used because the variable (normal or altered NPA) is nominal and the groups were unpaired (SC and IC). Percentages were used to describe the groups (Table 5).

**Table 5:** Descriptive values and comparison between groups concerning NPA results

Group	NPA			*x*^2^	*p*
	Normal	Altered	Total		
SC	12	27	39	4.93	0.026
	30.76%	69.23%	100%		
IC	16	10	26		
	61.53%	38.46%	100%		
Total	28	37	65		
	43.07%	56.92%	100%		

Source: Created by the author, 2025. *NPA* neuropsychological assessment, *SC* syndromic craniosynostosis, *IC* Isolated craniosynostosis

Group comparison of neuropsychological status (normal versus altered; coded as ≤ 25th percentile = below normative range) used chi-square methods for nominal unpaired groups, which are presented concisely in Table 5. Using corrected and internally consistent counts, altered NPA results were more frequent in the SC group (69.23%) than in the IC group (38.46%); overall, 56.92% of participants were classified as altered (odds ratio = 3.60). Neuropsychological outcomes differed significantly (*p* < 0.05) between groups: SC cases showed a higher prevalence of altered NPA results (69.23%; *n* = 27) than IC cases, a finding in line with the existing literature.

Cross-tabulation of pre–post data was available for 12 patients (6 SC, 6 IC) with a mean interassessment interval of 31.3 months (range 8–60 months). Given the small paired sample, analyses were descriptive and presented at the individual case level (Table 6). Case-level trajectories varied by the first available preoperative assessment with the first postoperative assessment (closest to surgery); a formal pre/post protocol was only implemented at the hospital in 2017, and variability in timing reflects high demand in the public service and patients’ geographic dispersion.

**Table 6:** Postoperative vs. preoperative neuropsychological test values

		Variables		NPA pre surgery		NPA post results		
N Patients	Age at pre-assessment (years;months)	Age at post-assessment (years)	Surgery technique	E	A	M	R	I
Patient 1	5 months	1	Cranioplasty	1	-	1	-	-
Patient 2	8 months	3	Cranioplasty	-	1	-	-	1
Patient 3	1;7	3;7	Cranioplasty	1	-	-	1	-
Patient 4	1;3	5;7	Posterior vault decompression	1	-	-	-	1
Patient 5	1;10	6;4	Cranioplasty	1	-	-	1	-
Patient 6	1;10	3;5	Posterior vault decompression	1	-	-	1	-
Patient 7	2;6	5;4	Cranioplasty	-	1	-	-	1
Patient 8	6;10	11;10	FFMA	-	1	1	-	-
Patient 9	8	9;6	FFMA	1	-	-	1	-
Patient 10	9	10;6	Cranioplasty w/distraction	-	1	-	1	-
Patient 11	11	13	Le Fort III and FFMA	1	-	-	1	-
Patient 12	15	17	Le Fort III	-	1	-	1	-
			Total	7	5	2	7	3

Source: Created by the author, 2025. *NPA* neuropsychological assessment, *FFMA* frontofacial monobloc advancement, *E* expected, *A* Altered, *M* maintained, *R* reduction, *I* increase

Cranioplasty demonstrated mixed outcomes; posterior vault decompression was associated with declines in several cases, Le Fort III tended toward maintenance or improvement in the older individuals observed, and FFMA cases showed poorer outcomes in this small series.

Overall, among the 12 paired cases, 3 improved, 3 maintained prior performance, and 6 declined; declines were more frequent in the IC subgroup. All scores were converted to percentiles, domains < 25 range were coded as “altered,” and were computed for each participant and descriptively examined in relation to age at assessment.

A weak negative correlation between age at surgery and degree of cognitive improvement was observed (*r* =  − 0.32), but this association was not statistically significant (*p* = 0.31), and the small paired sample precludes inferential conclusions about timing or technique effects.

## Discussion

To our knowledge, this study is among the first to present neuropsychological data on craniosynostosis within a Brazilian clinical cohort, which has implications for local practice and future research. The tools BDSGA, WASI, and CPM-RAVEN were selected to capture key developmental indices and to enable robust assessment of NPMD and intelligence across IC and SC groups; institutional policy mandates evaluation of all patients, but preoperative assessments were performed inconsistently.

Neuropsychological assessment revealed a higher prevalence of cognitive alterations in the SC group than in the IC group, consistent with prior reviews reporting that roughly half of the studies found FSIQ impairments in syndromic populations [28]. Syndromic cases—such as multiple-suture craniosynostosis—may present elevated intracranial pressure and substantial clinical and neurocognitive heterogeneity, factors that can adversely affect cognitive functioning [28].

Assessment of preserved functions revealed substantial intra-group variability. Early surgical intervention, particularly within the 9–12-month age range, has been suggested to mitigate these effects [3, 29].

Some IC cases exhibited cognitive alterations potentially related to lower median socioeconomic status; socioeconomic deprivation is associated with poorer developmental outcomes [30], and, as reported by Alperovich [31], school-age children treated for non-syndromic craniosynostosis typically exhibit subtle but reduced cognitive and behavioural performance.

According to the Brazilian Socioeconomic Classification [32] and the exchange rate on December 30th, 2024 (R$ 6.18 = US$ 1.00), most participants in this study came from families in the C class (approximately $913.61 to $2284/month) and E class (around $342.60/month). This suggests that their median socioeconomic status falls within the lower economic strata [33].

Conducted in a public health service in a low- and middle-income country (LMC), most participants (monthly incomes US$342.61–$913.60) relied on public healthcare; the mean age reflects heterogeneous service needs and underscores the need for policies guaranteeing appropriate, individualized care for children and adults.

It is noteworthy that some individuals in the IC group exhibited cognitive alterations. This finding can be attributed to socioeconomic status, particularly given that the median socioeconomic class of the participants was lower. Low economic conditions and parenting with low education levels significantly impact child development. Socioeconomic factors, such as neighbourhood deprivation and family affluence, are directly related to educational outcomes, cognition, mental health, and even resilience. Furthermore, research indicates that communication between brain regions via white matter is significantly linked to socioeconomic status, suggesting that childhood socioeconomic conditions can profoundly affect brain development [30].

Significant gross and fine motor impairments were observed across the sample, with a disproportionate burden in the IC group, suggesting a potential association between isolated craniosynostosis and motor deficits that warrants further investigation into underlying mechanisms. These findings are consistent with prior reports [5] of reduced VIQ, FSIQ, visuomotor integration, and motor-control measures in IC patients, even after surgical correction.

When assessed at younger ages, IC patients exhibited less pronounced motor impairments than SC patients; spatial construction matures with age, and visuospatial abilities support both motor and cognitive functions [34]. In contrast, Crouzon and Apert cases aged 10–33 all presented PIQ deficits, reflecting impaired visuospatial construction and visual cognition [22].

Although the SC group exhibited fewer motor impairments at younger ages, cognitive factors—particularly language—may nevertheless influence motor development in SC patients. Language and motor delays can persist after surgical correction and tend to become more evident from around age 6 with the onset of literacy demands [9, 34–36]; additionally, the COVID-19 pandemic has exacerbated language delays across both vulnerable and general populations, potentially worsening developmental outcomes [37].

Cranioplasty predominated in the cohort, while FFMA was performed infrequently—likely due to concerns about its risks despite yielding short-term midface correction without substantially altering underlying midface architecture or long-term craniofacial growth [38]. No clear association between technique and cognitive improvement was found, but earlier surgery favoured better outcomes; cranial-vault remodeling was associated with higher VIQ, PIQ, FSIQ, and improved visuomotor integration compared with spring-assisted surgery [7].

The lack of baseline NPA constrains evaluation of the procedure’s true long-term benefit, and postoperative school absence may further confound developmental outcomes. Assessment timing was uneven—80% (*n* = 52) were evaluated postoperatively, and 20% (*n* = 13) preoperatively—and many patients had undergone prior surgery elsewhere without prior NPA, accounting for their postoperative classification and limiting longitudinal interpretation.

## Conclusion

The results highlight cognitive challenges in craniosynostosis—particularly motor and communication impairments—with a higher prevalence in SC versus IC cases. Early, individualized rehabilitation can reduce developmental delays, although interindividual variability and syndrome-specific factors influence responsiveness. Precise NPA is essential to identify affected functions and preserved skills to guide targeted interventions. Observations relating to surgical technique and timing are descriptive and hypothesis-generating only; they do not establish causality given the small, heterogeneous subgroup and paired samples, underscoring the need for adequately powered prospective studies with standardized baseline assessments. Prolonged postoperative school absence may further worsen delays and warrants supported educational reintegration. Heterogeneity of assessment instruments limited comparability and may constrain generalizability.

## Limitations

Comparisons of motor and other neurodevelopmental outcomes are constrained by heterogeneous use of assessment instruments across age groups and by unequal representation of syndromic subtypes (for example, Apert and Crouzon) within age bands, which may influence observed rates of impairment; additionally, not all participants were assessed with the infant/preschool measures that provide gross motor scores.

## Supplementary Information

Below is the link to the electronic supplementary material.

ESM 1 Supplementary Material 1 (DOCX 28.6 KB)

ESM 2 Supplementary Material 2 (DOCX 29.3 KB)

## Data Availability

No datasets were generated or analysed during the current study.
