# Supplementary material for: Cognitive and neuropsychomotor development in craniosynostosis: an evaluation of the most affected functions
Source: Childs Nerv Syst. 2026 Jan 29;42(1):51. doi: 10.1007/s00381-026-07127-w (PMC12852296; doi:10.1007/s00381-026-07127-w)
Supplement: Supplementary file 1 — Supplementary Material 1 (DOCX 28.6 KB) [file 381_2026_7127_MOESM1_ESM.docx]

**Graph 1.** Graph with the distribution of patients with cognitive alterations across all tests between SC and IC group.

**Source:** Created by the author, 2025. **Legend:** GM- Gross motor; FM- Fine motor; AB- Adaptive Behaviour; L- Language; PS- Personal-social; FI- Fluid intelligence; FSIQ- Full-Scale Intelligence Quotient; PIQ- Performance Intelligence Quotient; VIQ- Verbal Intelligence Quotient.
