# Supplementary material for: Cognitive and neuropsychomotor development in craniosynostosis: an evaluation of the most affected functions
Source: Childs Nerv Syst. 2026 Jan 29;42(1):51. doi: 10.1007/s00381-026-07127-w (PMC12852296; doi:10.1007/s00381-026-07127-w)
Supplement: Supplementary file 2 — Supplementary Material 2 (DOCX 29.3 KB) [file 381_2026_7127_MOESM2_ESM.docx]

**Graph 2.** Distribution of age at first cranial surgery by diagnosis and suture type (N = 65).

**Legend:** CFND – Craniofrontonasal Dysplasia.
